# Saccadic inhibition in a guided saccade task

**DOI:** 10.7717/peerj.4493

**Published:** 2018-03-14

**Authors:** Isabel Dombrowe

**Affiliations:** Department of Cognitive Psychology: Judgment, Decision Making, Action, FernUniversität, Hagen, Germany; Department of Experimental Psychology, Otto-von-Guericke Universität Magdeburg, Magdeburg, Germany

**Keywords:** Saccadic inhibition, Main sequence, Eye movements

## Abstract

The eye movement system reacts very systematically to visual transients that are presented during the planning phase of a saccade. About 50 to 70 ms after the onset of a transient, the number of saccades that are started decreases, a phenomenon that has been termed saccadic inhibition. Saccades started just before this time window are hypometric compared to regular saccades, presumably because the presentation of the transient stops them in mid-flight. Recent research investigating the properties of repeated saccades to fixed locations found that these early saccades were additionally faster than expected from the main sequence relation, suggesting that a saccadic dead time during which saccades can no longer be modified does not exist. The present study investigated the properties of saccades to random locations in a guided saccade task. As expected, early saccades starting just before the saccadic inhibition dip in frequency were hypometric. Their velocity profiles implied that these saccades were actively stopped after reaching peak velocity. However, the peak velocities of these saccades did not generally deviate from the main sequence relation. The question whether an active stop of early saccades is incompatible with the idea of a saccadic dead time is open to debate.

## Introduction

Saccades are stereotyped and ballistic eye movements that re-align the fovea with objects in the visual field. There is a fixed relationship between the peak velocity of a saccade and its amplitude that has been termed the *main sequence* (Bahill, Clark & Stark, 1975; Zuber, Stark & Cook, 1965). The larger the amplitude of a saccade, the faster the peak velocity. For small saccades with an amplitude of less than 15° the increase in peak velocity is linear. For larger saccades, peak velocities increase only slightly until they reach a plateau at about 500–600 °/s.

Although the main sequence relation seems to hold for most saccades, some deviations from the main sequence have been reported. Bahill & Stark (1975), for example, found that fatigue of the eye movement system lead to saccades that were slower than predicted by the main sequence relationship. Blinked saccades have also been found to be slower than saccades that did not include an eyeblink (Rottach et al., 1998; Rambold, Sprenger & Helmchen, 2002).

It has been shown that the eye movement system reacts in a very systematic manner to visual transients, such as flashes, that are presented during the planning phase of a saccade. Reingold & Stampe (1999, 2002) asked their participants to make saccades to targets in the visual field and perturbed some of these saccades with very brief visual transients. They found that only very few saccades were started 50–70 ms after the presentation of the transient, creating a characteristic dip in the histogram of saccadic frequencies. This effect is called *saccadic inhibition (SI)* and is usually followed by a rebound phase where the omitted saccades are excuted with a delay. Saccadic inhibition has also been found for microsaccades (*microsaccadic inhibition*; White & Rolfs, 2016; Engbert, 2012).

Recently, Buonocore, McIntosh & Melcher (2016) made an interesting observation regarding the peak speed of saccades that were started briefly after the presentation of a visual transient. In three different experiments, they presented brief (20 ms) flashes at varying points in time before their participants made a saccade from the center of the screen to a target at up to three different eccentricities. As expected, they found saccadic inhibition from about 50 ms to 90 ms after flash onset. Moreover, they found that saccades that were perturbed by a flash were shorter than saccades that were not perturbed by a flash, if they had been started just before the SI dip. This effect has been observed before (Edelman & Xu, 2009; Guillaume, 2012). In two of four conditions, they found that these hypometric saccades had higher peak velocities than saccades that were not perturbed by a flash. These hypometric and fast saccades occurred very early, already at around 20 ms to 40 ms after flash onset. A similar result has recently been obtained for microsaccades (Buonocore et al., 2017).

According to the authors, this result goes against the assumption that there is a *point of no return* followed by a *saccadic dead time*, when the programming of the saccade is complete and can no longer be altered (Ludwig, Mildinhall & Gilchrist, 2007). Although the actual time span covered by the *saccadic dead time* has been found to vary, a span of 20 ms to 40 ms would be too brief to allow for a complete processing chain from flash perception and the modification of the saccade plan to the activation of the eye muscles. Buonocore, McIntosh & Melcher (2016) argue that the higher peak velocity of saccades started briefly after a visual transient, but before the saccadic inhibition dip, constitutes a “violation of the main sequence” (e.g., pages 575 and 576). They also argue that the peak velocities of these saccades show that saccades can still be modified during the *saccadic dead time*, and hence, that a *saccadic dead time* does not exist.

The first aim of the present study was to test, whether these findings can be replicated in a guided saccade task. In contrast to the task used by Buonocore, McIntosh & Melcher (2016), the target locations in a guided saccade task are not predictable for the observer. Since the pacing in a guided saccade task is faster than in a trial based experiment, two conditions were tested. The first kept the original timing of the study by Buonocore, McIntosh & Melcher (2016). In the second condition, a new saccade target appeared as soon as the saccade to the previous target was completed.

The second aim of the study was to see whether any peak velocity differences from the main sequence are enhanced by using a saccade detection algorithm that uses an adaptive threshold to determine the onset of a saccade. The built-in saccade detection algorithm of the Eyelink 1000 eyetracker, that was used by Buonocore, McIntosh & Melcher (2016) and in the present study detects saccades based on fixed velocity and accelaration thresholds for saccade onsets. The adaptive algorithm used in the present study (Nyström & Holmqvist, 2010), calculates an individual saccade onset threshold based on the noise found in the data. This means that, among other things, the same saccades are detected earlier in time. Fitting an exponential function (i.e., main sequence fit) to the amplitudes and peak velocities of the saccades detected by the Nyström & Holmqvist (2010) algorithm often results in a better fit than for saccades detected using the built-in algorithm of the eye tracker.

The third aim of this study was to evaluate, whether the higher peak velocities of saccades whose saccadic dead time included the presentation of a visual transient should be termed a “violation of the main sequence” and whether this would be incompatible with the idea of a saccadic dead time.

Participants performed two conditions of a guided saccade task. The first condition had a very similar timing to the experiments reported in Buonocore, McIntosh & Melcher (2016), that is, a new saccade target was presented after a delay of 1 s, while the participants were required to hold fixation. In the second condition, this delay was reduced to 100 ms, resulting in a fast scanning of the display. In both conditions, one third of all saccades were perturbed by a flash presented 150 ms after the onset of a new saccade target.

## Methods

### Participants, design and procedure

Nineteen volunteers (16 university students, three non-student volunteers) participated in the experiment. They all reported normal or corrected to normal vision. None of the participants had any neurological disorders. All participants signed a consent form before the start of the experiment. The study was conducted in compliance with the Declaration of Helsinki, except that the study was not preregistered. The University of Magdeburg granted ethical approval to carry out the study within its facilities (BA Grimm/062017).

The experiment was implemented using the Psychophysics Toolbox (Brainard 1997) for Matlab (Mathworks, Natick, MA, USA) and run on a Macbook laptop computer (2 GHz CPU, 2 GB RAM) with a 24-inch monitor operating at 60 Hz and a resolution of 1,920 × 1,080 pixels. Viewing distance was 85 cm and was maintained with a chinrest. Eye movements of the left eye were recorded at 1,000 Hz with an Eyelink 1000 eye tracker (SR research, Ontario, Canada), using the built-in saccade detection algorithm with a velocity threshold of 30 °/s and an acceleration threshold of 9,500 °/s^2^ for the first data analysis and the adaptive saccade detection algorithm of Nyström & Holmqvist (2010) for the second data analysis.

The background color of the monitor was set to a medium gray (RGB 128,128,128), all fixation and saccade targets were black (RGB 0,0,0), light flashes were white (RGB 255,255,255). The room was dimly illuminated by a background light (0.5 lx).

**Figure 1:**
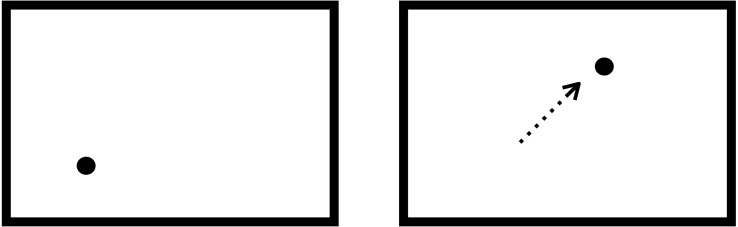
Schematic depiction of the stimuli used in the experiment. A small dot appeared in a random position on the screen. Participants had to fixate this dot for 1,000 ms in the *slow* condition and for 100 ms in the *fast* condition until the dot disappeared and a new dot appeared between 5 and 15° from the first dot. Participants were asked to follow the dot with their eyes. In one third of all trials, the top and the bottom 100 px of the monitor were switched to white for the duration of one frame (flash) starting 150 ms after the onset of the new dot.

The task was a *visually guided saccade* task as depicted in Fig. 1. At the start of the experiment, a small dot with a radius of 0.4° appeared in a random position on the screen. Participants were asked to fixate this dot until a second, identical, dot appeared and the first dot disappeared. The distance between the two dots was randomly chosen between 5 and 15° of visual angle. The participants were instructed to make a saccade to the second dot as soon as it appeared on the screen.

There were two conditions, tested in two separate blocks. In the first condition, the required fixation duration on the first dot was 1,000 ms until the second dot appeared (*slow* condition). In the second condition, the required fixation duration was 100 ms (*fast* condition). Each condition consisted of 900 guided saccades. One third of these guided saccades were perturbed by a flash. 150 ms after the second dot appeared, the top and bottom 100 pixels (1.9°) from the upper and lower border of the screen were switched to white for the duration of one frame (16.7 ms). The dots never appeared in this margin. Saccades with flash were chosen randomly. The first dot disappeared together with the onset of the flash or after 150 ms after the presentation of the second dot.

Each condition consisted of six blocks of 150 guided saccades. A 13-point calibration was conducted at the start of each block. Half of the participants started with the fast condition, the other half with the slow condition. With frequent breaks for the participant the experiment took about 1 h.

### Data analysis

Only the first saccade after the onset of the second dot was included in the analysis, yielding exactly 600 *no-flash saccades* and 300 *flash saccades* for each condition with the built-in algorithm of the eye tracker and maximally five missed saccades per participant with the adaptive algorithm.

Saccadic *latency* was defined as the time span between the onset of the second dot and the start of the first saccade after this onset. The figures in the Results section depict the latency relative to the offset of the flash, resulting in a minimum latency value of −167 ms, corresponding to the onset of the second dot.

Saccadic *gain* was computed by dividing the amplitude of the saccade by the center-to-center distance between the first and the second dot.

*Normalized peak velocity* was computed by dividing the peak velocity of the saccade by the value on the main sequence fit to the peak velocities below 800 °/s and amplitudes below 30° of the *no flash* condition as a function of amplitude.

For all analyses reported in the next sections, saccadic latencies from 0 to 400 ms were binned in 20 bins, each containing a time span of 20 ms. To compute the normalized frequency, saccades in each bin were counted and normalized such that the area below the resulting curve was equal to 1. Saccadic gains and normalized peak velocities of each bin were averaged by computing the median value of each bin.

Average flash and no-flash saccades values of each bin were compared using paired, two-sided *t*-tests that were Bonferroni corrected for multiple comparisons. Analyses of variance (ANOVAs) were conducted in R. The data of the this study is available at the Open Science Framework project website (DOI: 10.17605/OSF.IO/PVBWM).

#### The adaptive algorithm

The built-in algorithm of the Eyelink eyetracker parses *x*, *y* coordinates into saccades and fixations using fixed velocity and acceleration thresholds. In 2010, Nyström & Holmqvist published a simple algorithm, which detects saccades, glissades—small corrective saccades immediately following a saccade—and fixations. The algorithm can be applied on a trial-by-trial level, taking the individual noise-level of the trial into account. In the present study, the time from the onset of the second dot to 400 ms after its onset was substituted for one ‘trial’. In the following analysis, this algorithm was used with a peak threshold of 5× mean trial velocity and an onset threshold of 2.5× mean trial velocity (see Nyström & Holmqvist (2010) for a list of parameters). Prior to event detection, raw *x*, *y* coordinates were Savitsky-Golay filtered with a filter length of 19 ms. Minimum saccade duration was set to 9 ms, maximum saccade duration to 100 ms.

## Results

The first paragraph of each subsection reports the results obtained using the built-in saccade detection algorithm of the Eyelink 1000 eye tracker, followed by the results obtained with the adaptive algorithm of Nyström & Holmqvist (2010).

### Saccadic inhibition

The presentation of a brief flash induced saccadic inhibition, which was followed by a rebound phase, in both conditions and with both saccade detection algorithms. These findings reproduce the saccadic inhibition and rebound effect found in previous studies (e.g., Edelman & Xu, 2009; Reingold & Stampe, 2002; Buonocore, McIntosh & Melcher, 2016).

#### Eyelink algorithm

**Figure 2:**
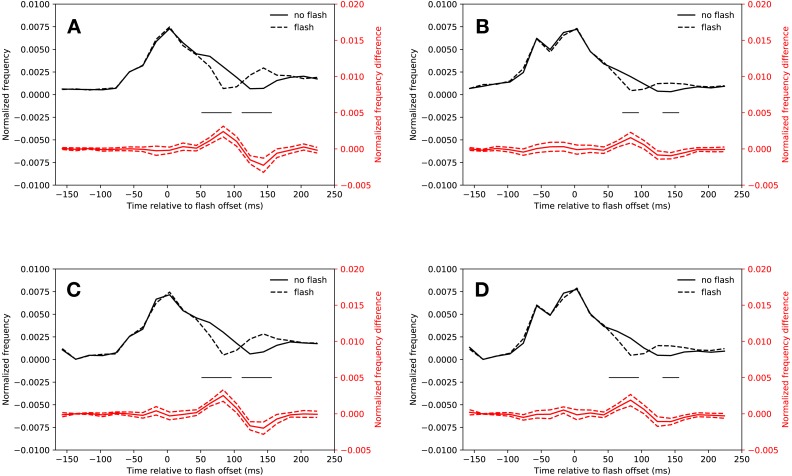
Saccadic inhibition. Red lines show the difference between *flash saccacades* and *no-flash saccades* together with a 95% confidence interval for this difference. Black lines indicate a signficant difference after correcting for multiple comparisons (*p* < 0.0025). (A) Eyelink algorithm: *Slow* condition. (B) Eyelink algorithm: *Fast* condition. (C) Adaptive algorithm: *Slow* condition. (D) Adaptive algorithm: *Fast* condition.

Figure 2A shows the normalized saccadic frequency in the *slow* condition. Participants made significantly less saccades whenever a flash was presented in the latency bins starting from 54 ms after flash offset and ending 94 ms after flash onset (*t*(18) = 5.07, *p* < 0.001, *t*(18) = 6.62, *p* < 0.001). A rebound occurred in the latency bins starting from 114 ms and ending 154 ms after flash offset (*t*(18) = 5.45, *p* < 0.001, *t*(18) = 4.70, *p* < 0.001; the interaction of saccade condition (flash, no-flash) and latency bin (20 bins) of a repeated measures ANOVA was significant: *F*(19, 342) = 14.46, *p* < 0.001). All *t*-tests were Bonferroni corrected for multiple comparisons (20 tests, *p* < 0.0025). All *t*-tests reported below were corrected in this way.

Figure 2B depicts the normalized saccadic frequency in the *fast* condition. The results were almost identical to the *slow* condition. Participants made less saccades whenever a flash was presented in the latency bin starting from 74 ms and ending at 94 ms (*t*(18) = 4.33, *p* < 0.001). A rebound occurred in the latency bin from 134 ms to 154 ms (*t*(18) = 4.44, *p* < 0.001, the interaction of saccade condition (flash, no-flash) and latency bin (20 bins) was significant: *F*(19, 342) = 5.252, *p* < 0.001).

#### Adaptive algorithm

As Fig. 2C depicts, saccadic inhibition and rebound of the *slow* condition could be reproduced using the adaptive saccade detection algorithm. There were fewer saccades with latencies from 54 ms to 114 ms after flash onset than in the same time bins when no flash was presented (*t*(18) = 7.67, *p* < 0.001, *t*(18) = 6.49, *p* < 0.001, *t*(18) = 4.08, *p* = 0.00070). There was a rebound from 114 ms to 154 ms (*t*(18) = 5.94, *p* < 0.001, *t*(18) = 4.78, *p* = 0.00016, significant interaction of saccade condition (flash, no-flash) with latency bin (20 bins): *F*(19, 342) = 15.23, *p* < 0.001).

This was also the case for the *fast* condition (see Fig. 2D). Saccadic inhibition lasted from 54 ms to 94 ms (*t*(18) = 4.19, *p* < 0.001, *t*(18) = 4.91, *p* < 0.001). A rebound was found from 134 ms to 154 ms (*t*(18) = 4.97, *p* < 0.001; interaction of saccade condition (flash, no-flash) with latency bin (20 bins): *F*(19, 342) = 8.07, *p* < 0.001).

### Gain

Saccades that started in the time span between the offset of the flash and the saccadic inhibition dip were clearly shorter than regular saccades that started around the same time. The latency bin in which this effect was first observed was slightly different for the *slow* and the *fast* condition, but earlier than the *point of no return* that has been found at about 60 ms before saccade onset (Ludwig, Mildinhall & Gilchrist, 2007), indicating that the flash fell in to the saccadic dead time of these hypometric saccades.

#### Eyelink algorithm

**Figure 3:**
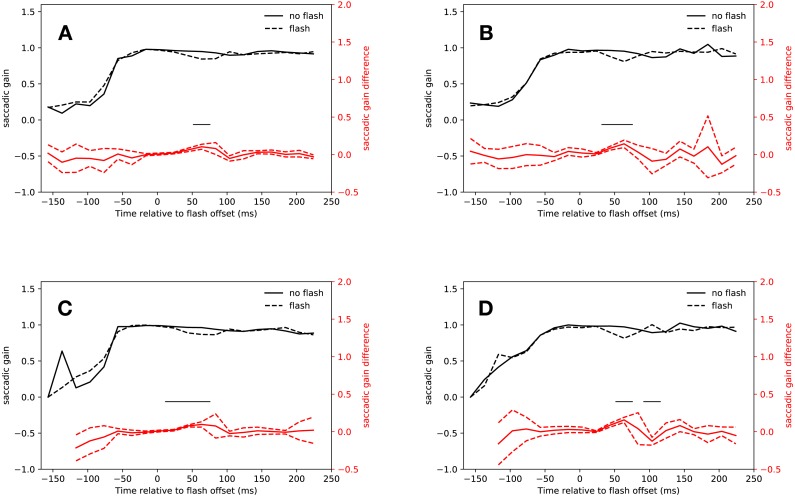
Gain. Red lines show the difference between *flash saccacades* and *no-flash saccades* together with a 95% confidence interval for this difference. The adaptive algorithm did not assign a sufficient number of saccades to the first two latency bins. Black lines indicate a signficant difference after correcting for multiple comparisons (*p* < 0.0025). (A) Eyelink algorithm: *Slow* condition. (B) Eyelink algorithm: *Fast* condition. (C) Adaptive algorithm: *Slow* condition. (D) Adaptive algorithm: *Fast* condition.

As Fig. 3A shows, saccades in the *slow* condition that started from 14 ms after flash offset were clearly hypometric relative to *no-flash saccades* (14 ms–34 ms: *t*(18) = 3.94, *p* < 0.001, 34 ms–54 ms: *t*(18) = 5.74, *p* < 0.001). This effect extended into the first bin of the saccadic inhibition dip (54 ms–74 ms: *t*(18) = 6.45, *p* < 0.001; interaction of saccadic condition (flash, no-flash) with latency bin (−27 ms to 74 ms relative to flash offset): *F*(4, 72) = 18.19, *p* < 0.001).

Again, the results for the *fast* condition were very similar (see Fig. 3B). Saccades that started just before the saccadic inhibition dip were hypometric relative to *no-flash saccades* (34 ms–54 ms: *t*(18) = 6.71, *p* < 0.001, 54 ms–74 ms: *t*(18) = 5.89, *p* < 0.001; interaction of saccade condition with latency bin (−27 ms to 74 ms relative to flash offset): *F*(4, 72) = 8.014, *p* < 0.001).

#### Adaptive algorithm

The analysis of the saccade amplitudes in both conditions using the adaptive algorithm for saccade detection were very similar to those obtained with the built-in algorithm of the eye tracker (see Figs. 3C and 3D). *Flash saccades* were hypometric relative to *no-flash saccades* from 14 ms to 74 ms after flash offset (*t*(18) = 4.08, *p* < 0.001, *t*(18) = 11.63, *p* < 0.001, *t*(17) = 5.52, *p* < 0.001; interaction of saccade condition with latency bin (−27 ms to 54 ms relative to flash offset): *F*(3, 54) = 35.61, *p* < 0.001).

**Table 1:** Statistical comparison of saccadic gain in the *slow* condition after the presentation of a flash and without flash. Bolded text indicates saccadic inhibition/rebound found with both algorithms. Italicized text indicates that only one of the algorithms found saccadic inhibition/rebound.

Saccadic gain in the *slow* condition							
Bin center	Eyelink			Adaptive			Comments
	*t*-statistic	df	*p*-value	*t*-statistic	df	*p*-value	
−157 ms	0.33	13	0.74719	–			
−137 ms	1.54	13	0.14812	–			
−117 ms	0.54	11	0.60232	2.58	14	0.02198	
−97 ms	1.02	14	0.32781	1.45	15	0.16786	
−77 ms	1.02	13	0.32752	0.97	16	0.34840	
−57 ms	0.23	15	0.82448	0.47	14	0.64485	
−37 ms	1.01	17	0.32590	0.85	18	0.40620	
−17 ms	0.14	18	0.89203	0.81	18	0.43091	
3 ms	1.25	18	0.22571	1.76	18	0.09494	
24 ms	3.94	18	0.00096	4.08	18	0.00071	Hypometric saccades
44 ms	5.47	18	<0.0001	11.63	18	<0.0001	Hypometric saccades
**64 ms**	**6.45**	**18**	**<0.0001**	**5.52**	**17**	**<0.0001**	**Hypometric saccades**
**84 ms**	**2.11**	**15**	**0.05232**	**0.97**	**17**	**0.34445**	
*104 ms*	*2.93*	*17*	*0.00943*	*1.66*	*17*	*0.11625*	
**124 ms**	**0.10**	**17**	**0.92139**	**0.33**	**16**	**0.74418**	
**144 ms**	**3.13**	**18**	**0.00579**	**0.52**	**18**	**0.61268**	
164 ms	2.52	17	0.02200	0.16	17	0.87264	
184 ms	0.16	16	0.87218	0.62	16	0.54335	
204 ms	0.60	15	0.55862	0.19	15	0.85101	
224 ms	2.35	17	0.03133	0.23	16	0.81733	

In the *fast* condition, saccades that were perturbed by a flash were hypometric relative to *no-flash saccades* from 34 ms to 74 ms after flash offset (*t*(18) = 6.91, *p* < 0.001, *t*(18) = 9.32, *p* < 0.001, interaction of saccade condition (flash, no-flash) with latency bin (−27 ms to 54 ms relative to flash offset): *F*(4, 72) = 18.73, *p* < 0.001). Both algorithms consistently found hypometric saccades in the latency bin from 34 ms to 54 ms after flash offset.

Tables 1 and 2 show the results obtained with both algorithms.

**Table 2:** Statistical comparison of saccadic gain in the *fast* condition after the presentation of a flash and without flash. Bolded text indicates saccadic inhibition/rebound found with both algorithms. Italicized text indicates that only one of the algorithms found saccadic inhibition/rebound.

Saccadic gain in the *fast* condition							
Bin center	Eyelink			Adaptive			Comments
	*t*-statistic	df	*p*-value	*t*-statistic	df	*p*-value	
−157 ms	0.54	15	0.59857	–			
−137 ms	0.24	17	0.81134	–			
−117 ms	0.92	16	0.37141	1.19	13	0.25492	
−97 ms	0.54	18	0.59611	0.09	16	0.93197	
−77 ms	0.01	18	0.99446	0.48	16	0.64059	
−57 ms	0.15	18	0.88065	0.02	18	0.98436	
−37 ms	1.02	18	0.31971	0.80	18	0.43543	
−17 ms	1.69	18	0.10880	1.47	18	0.15962	
3 ms	0.77	18	0.45314	1.31	18	0.20692	
24 ms	1.21	18	0.24076	0.24	18	0.81502	
44 ms	6.71	18	<0.0001	6.91	18	<0.0001	Hypometric saccades
*64 ms*	*5.89*	*18*	*<0.0001*	*9.32*	*18*	*<0.0001*	*Hypometric saccades*
**84 ms**	**0.76**	**11**	**0.46471**	**0.40**	**12**	**0.69818**	
104 ms	1.09	13	0.29689	5.01	13	0.00024	
124 ms	1.62	13	0.12962	0.31	15	0.76470	
**144 ms**	**1.51**	**9**	**0.16557**	**2.12**	**15**	**0.05721**	
164 ms	0.46	13	0.65637	0.02	12	0.98502	
184 ms	0.52	14	0.61053	0.59	12	0.56616	
204 ms	2.41	11	0.03470	0.17	12	0.86491	
224 ms	0.28	14	0.78345	0.94	14	0.36209	

### Peak velocity

**Figure 4:**
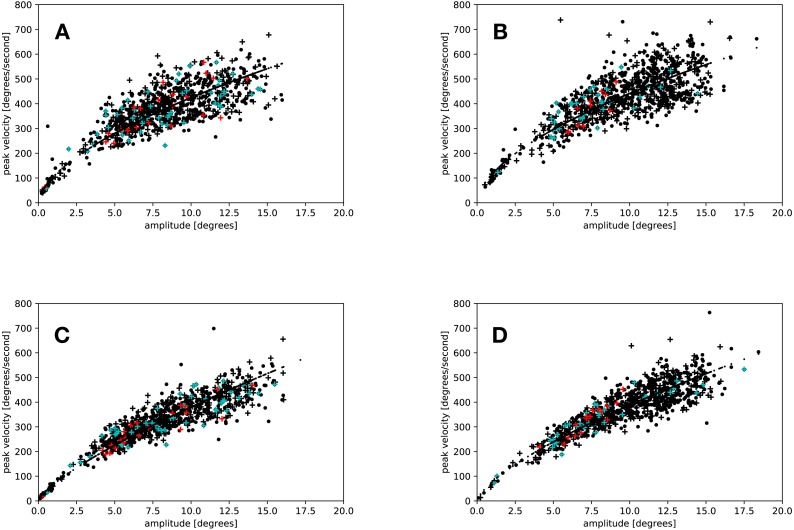
Main sequences for the saccades of a random participant. Black crosses and dots represent saccades in the flash and the no-flash condition. Red crosses indicate saccades that were started in the time window from 34 ms to 54 ms after flash offset; blue crosses indicate regular saccades started in the same time window. (A) Eyelink algorithm: *Slow* condition. (B) Eyelink algorithm: *Fast* condition. (C) Adaptive algorithm: *Slow* condition. (D) Adaptive algorithm: *Fast* condition.

A comparison of the main sequence fits showed that the amplitudes and peak velocities obtained by the adaptive algorithm lead to a better fit in the *slow* (eyelink average *r*^2^ = 0.87, adaptive average *r*^2^ = 0.95, *t*(18) = 4.305, *p* < 0.001), but not in the *fast* condition (eyelink average *r*^2^ = 0.93, adaptive average *r*^2^ = 0.92, *t*(18) = 0.339, *p* = 0.738). Figure 4 depicts the main sequences for a random participant of the present study.

Results similar to those of Buonocore, McIntosh & Melcher (2016) were only found in the *fast* condition when the adaptive algorithm was used for saccade detection.

#### Eyelink algorithm

Neither the hypometric saccades launched before the saccadic inhibition dip, nor the few saccades started during saccadic inhibition were faster than regular saccades, although there was a non-significant trend for saccades in both conditions that started between 34 ms and 54 ms after flash offset (*slow* condition: 14 ms–34 ms: *t*(18) = 0.67, *p* = 0.5096, 34 ms–44 ms: *t*(18) = 2.92, *p* = 0.0092, 44 ms–54 ms: *t*(18) = 1.32, *p* = 0.2037, the interaction of condition and latency bin was not significant: *F*(4, 72) = 1.244, *p* = 0.3000; *fast* condition: 34 ms–54 ms: *t*(18) = 2.96, *p* = 0.0083, 54 ms–74 ms: *t*(18) = 0.95, *p* = 0.3562, the interaction of condition and latency bin was not significant: *F*(4, 72) = 1.923, *p* = 0.116).

**Figure 5:**
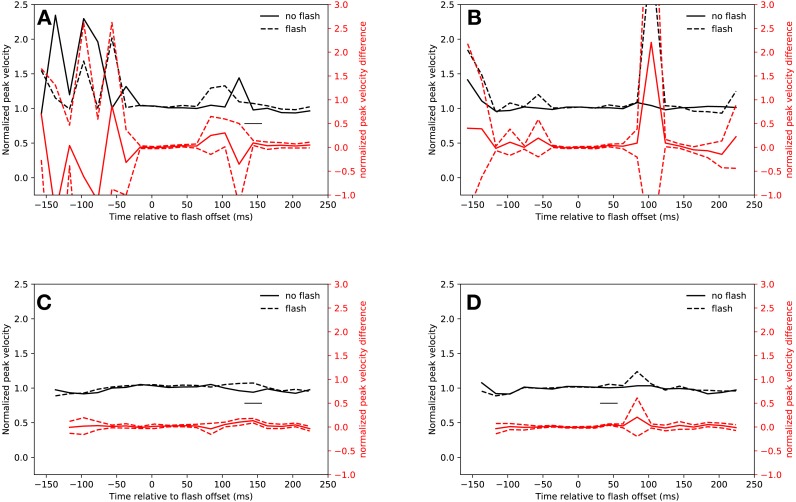
Peak velocity. Red lines show the difference between *flash saccacades* and *no-flash saccades* together with a 95% confidence interval for this difference. The adaptive algorithm did not assign a sufficient number of saccades to the first two latency bins. Black lines indicate a signficant difference after correcting for multiple comparisons (*p* < 0.0025). (A) Eyelink algorithm: *Slow* condition. (B) Eyelink algorithm: *Fast* condition. (C) Adaptive algorithm: *Slow* condition. (D) Adaptive algorithm: *Fast* condition.

Saccades in the rebound phase of the *slow* condition were significantly faster when a flash had been presented than saccades of trials without flash during the same latency bin (134 ms–154 ms: *t*(18) = 3.68, *p* = 0.0017). The peak velocity difference between *flash saccades* and *no-flash saccades* in the same rebound latency bin of the *fast* condition was not significant (*t*(9) = 1.36, *p* = 0.2065, after exclusion of data of participants, who did not make any saccades in this time bin). Figures 5A and 5B show the peak velocities for the *slow* and *fast* conditions, respectively.

#### Adaptive algorithm

Figures 5C and 5D show the saccadic peak velocity for the *slow* and *fast* conditions. Saccades that were started just before the saccadic inhibition dip in the *slow* condition were not faster than regular saccades (14 ms–34 ms: *t*(18) = 2.65, *p* = 0.0164, 34 ms–54 ms: *t*(18) = 2.68, *p* = 0.0154, 54 ms–74 ms: *t*(17) = 0.1941, the interaction of condition and latency bin was not significant: *F*(3, 54) = 1.42, *p* = 0.247). However, this was the case in the *fast* condition (34 ms–54 ms: *t*(18) = 6.83, *p* < 0.0001, 54 ms–74 ms: *t*(18) = 1.24, *p* = 0.2309, the interaction of condition and latency bin was significant: *F*(3, 54) = 11.98, *p* < 0.001).

Saccades from 134 ms to 154 ms after flash offset in the *slow* condition had a higher peak velocity than saccades in the same time bin that were not preceded by a flash (*t*(18) = 5.73, *p* < 0.0001). This was not the case in the *fast* condition (*t*(11) = 0.88, *p* = 0.3976).

Tables 3 and 4 list the results obtained with both algorithms.

**Table 3:** Statistical comparison of the peak velocities of saccades in the *slow* condition after the presentation of a flash and without flash. Bolded values indicate time bins with hypometric saccades.

Peak velocity in the *slow* condition							
Bin center	Eyelink			Adaptive			Comments
	*t*-statistic	df	*p*-value	*t*-statistic	df	*p*-value	
−157 ms	1.51	13	0.15533	–			
−137 ms	1.07	13	0.30392	–			
−117 ms	0.20	11	0.84934	0.10	14	0.92216	
−97 ms	0.39	14	0.70032	0.22	15	0.82276	
−77 ms	1.36	13	0.19643	0.69	16	0.49876	
−57 ms	1.03	17	0.31934	0.92	14	0.37327	
−37 ms	0.94	17	0.35899	1.05	18	0.30622	
−17 ms	0.46	18	0.65281	0.94	18	0.35974	
3 ms	0.50	18	0.62236	0.67	18	0.50997	
**24 ms**	**0.67**	**18**	**0.50959**	**2.65**	**18**	**0.01643**	
**44 ms**	**2.92**	**18**	**0.00916**	**2.68**	**18**	**0.01538**	
**64 ms**	**1.32**	**18**	**0.20369**	**1.35**	**17**	**0.19414**	
84 ms	1.30	15	0.21209	0.68	17	0.50467	
104 ms	2.14	17	0.04799	2.23	17	0.03920	
124 ms	0.85	17	0.40958	3.04	16	0.00774	
144 ms	3.68	18	0.00171	5.73	18	<0.0001	Higher peak velocity
164 ms	0.94	17	0.35903	0.90	17	0.38329	
184 ms	1.70	16	0.10791	0.55	16	0.58856	
204 ms	1.43	15	0.17413	2.35	15	0.03288	
224 ms	1.71	17	0.10554	1.41	16	0.17896	

**Table 4:** Statistical comparison of the peak velocities of saccades in the *fast* condition after the presentation of a flash and without flash. Bolded values indicate time bins with hypometric saccades.

Peak velocity in the *fast* condition							
Bin center	Eyelink			Adaptive			Comments
	*t*-statistic	df	*p*-value	*t*-statistic	df	*p*-value	
−157 ms	0.47	15	0.64641	–			
−137 ms	0.79	17	0.44126	–			
−117 ms	0.92	16	0.37453	0.66	13	0.52010	
−97 ms	0.81	18	0.43008	0.31	17	0.76231	
−77 ms	0.10	18	0.92067	0.26	17	0.79973	
−57 ms	1.02	18	0.32394	0.15	18	0.88120	
−37 ms	1.91	18	0.07286	2.07	18	0.05369	
−17 ms	1.41	18	0.17682	1.01	18	0.32785	
3 ms	0.19	18	0.85161	0.92	18	0.37122	
24 ms	0.41	18	0.68869	0.31	18	0.75725	
**44 ms**	**2.96**	**18**	**0.00834**	**6.83**	**18**	**<0.0001**	**Higher peak velocity**
**64 ms**	**0.95**	**18**	**0.35616**	**1.24**	**18**	**0.23089**	
84 ms	0.65	11	0.53238	1.07	12	0.30591	
104 ms	1.07	13	0.30571	1.06	13	0.30617	
124 ms	2.45	13	0.02899	0.70	15	0.49408	
144 ms	1.36	9	0.20647	0.88	11	0.39759	
164 ms	1.63	13	0.12670	0.03	12	0.97905	
184 ms	1.01	14	0.33119	2.35	13	0.03542	
204 ms	1.09	11	0.29746	1.29	13	0.21895	
224 ms	0.70	14	0.49563	0.40	14	0.69570	

### Velocity profiles

A closer look at Tables 1–4 and the Figures shows that there are two time windows that warrant further inspection. *Flash saccades* that were started between 34 ms and 54 ms after flash offset were found to be hypometric relative to *no-flash saccades*, irrespective of experiment timing and saccade detection algorithm.

There was trend towards faster *flash saccades* in comparison to *no-flash saccades* in the *slow* condition (see Table 3, 34 ms to 54 ms). The flash saccades started during this time window in the *fast* condition were indeed faster than regular saccades (see Table 4, 34 ms to 54 ms), when computed using the adaptive algorithm. This is approximately the time window during which Buonocore, McIntosh & Melcher (2016) found faster hypometric saccades (34–54 ms after flash offset corresponds to approximately 17 ms to 37 ms after flash onset).

The second time window of interest was the rebound phase from 134 ms to 154 ms after flash offset. *Flash saccades* that started in the rebound phase were faster than *no-flash saccades* in the same time window in the *slow* condition. Further analysis will start with the latter time window.

**Figure 6:**
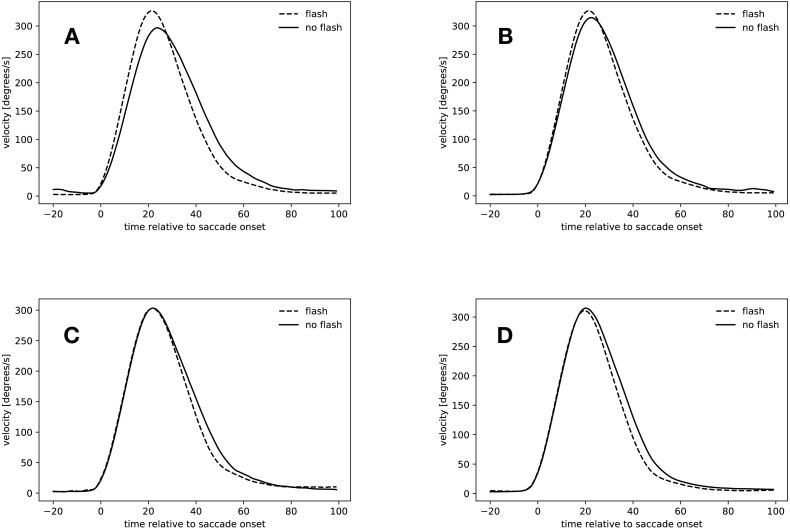
Velocity profiles. (A) Saccades started in the rebound phase. (B) *Flash saccades* started during the rebound phase compared to *no-flash saccades* started during saccadic inhibition. (C) *Slow* condition: 34 ms to 54 ms after flash offset. (D) *Fast* condition: 34 ms to 54 ms after flash offset.

#### Rebound saccades (134 ms–154 ms after flash offset)

Figure 6A shows the velocity profiles for the rebound saccades in the *slow* condition. Velocity profiles for this time window were only computed for the *slow* condition, since not all participants started saccades during that time in the *fast* condition (see Table 4). In the *slow* condition, *flash saccades* peaked 21 ms after saccade onset, *no-flash saccades* 24 ms after saccade onset. The maximal difference in velocity occurred 16 ms after saccade onset, where *flash saccades* were 51 ms faster than *no-flash saccades* (*t*(18) = 4.505, *p* = 0.0003).

These results confirm the finding that rebound saccades had a tendency to be faster than regular saccades in the same time window. However, the question arises, if this comparison actually compared the right saccades. After all, rebound saccades are those saccades that were suppressed during saccadic inhibition. Figure 6B suggests that this might not be the case. It depicts the velocity profiles for *flash saccades* in the rebound phase from 134 ms to 154 ms after flash offset and those of *no-flash saccades* in the time window of maximal saccadic inhibition from 74 ms to 94 ms after flash offset in the *slow* condition. Both saccade types peaked at about the same time after saccade onset (21 ms *flash saccades* during the rebound phase, 22 ms during saccadic inhibition), peak velocity did not differ, however (*t*(18) = 1.53, *p* = 0.1436). A comparison of normalized peak velocities of *flash saccades* in the rebound phase (134 ms to 154 ms after flash offset, mean normalized velocity = 1.07) and *no-flash saccades* in the maximum saccadic inhibition time window (74 ms to 94 ms after flash offset, mean normalized peak velocity = 1.05) confirmed this finding (*t*(18) = 1.537, *p* = 0.1417). Apparently, the peak velocity difference during the rebound phase was not caused by an increase in *flash saccade* peak velocity (74 ms to 94 ms vs. 134 ms to 154 ms: *t*(17) = 0.971, *p* = 0.3450), but rather by a peak velocity decrease of the *no-flash saccades* (74 ms to 94 ms vs. 134 ms to 154 ms, 1.05 to 0.94: *t*(18) = 5.42, *p* < 0.001, see also Fig. 5C).

#### Early hypometric saccades (34 ms–54 ms after flash offset)

Figures 6C and 6D depict the velocity profiles of saccades with latencies between 34 ms and 54 ms after flash offset. In the *slow* condition, both *flash saccades* and *no-flash saccades* peaked at 22 ms after flash offset, however, velocities did not differ significantly at this point in time (*t*(18) = 0.004, *p* = 0.9965). From 20 ms to 79 ms after flash offset, *flash sacccades* were slower than *no-flash saccades*, indicating that they were decelerating faster (*t*(18) = 3.137, *p* = 0.0057).

*Flash saccades* and *no-flash saccades* in the *fast* condition peaked at 20 ms after flash offset. Their peak velocities did not differ (*t*(18) = 0.530, *p* = 0.6027). From 17 ms to 100 ms after flash offset, *flash saccades* were slower than *no-flash saccades* (*t*(18) = 4.678, *p* = 0.0002), again indicating that these saccades decelerated faster than non-perturbed saccades in the same time window.

Apparently, saccades that started between 34 ms and 54 ms after flash offset were accelerating regularly until reaching peak velocity. Saccades that were perturbed by a flash then decelerated faster than regular saccades. Thus, early hypometric saccades seem to have been actively stopped in mid-flight. This stop has to be an active process, since the velocity of both saccade types is identical until reaching peak velocity. If the eye now passively decelerated in both conditions, the velocity profiles would remain identical until the end of the saccade.

## Discussion

The aim of the present study was to investigate the properties of saccades that are started after the presentation of a flash, but before the characteristic dip in saccadic frequency that typically starts about 60 ms after this flash (*saccadic inhibition*). Since the flash falls into the saccadic dead time of these saccades, any differences from saccades that were not perturbed by a flash and started at the same time potentially question the existence of a saccadic dead time.

The present study showed that the early saccades that started after, but not during the flash, were hypometric. However, these saccades did not generally have different peak velocities. An analysis of the velocity profiles of these early, hypometric saccades revealed that they accelerated just as saccades that were not perturbed by a flash, but decelerated faster, leading to a shorter duration and thus amplitude.

Moreover, the saccades during the rebound phase were found to have a faster peak velocity than regular saccades started at the same point in time. These rebound saccades also showed a non-significant trend towards hypometry. However, these saccades were not significantly faster than regular saccades that were started when saccades perturbed by a flash were in the saccadic inhibition phase.

Hypometric saccades just prior to the saccadic inhibition phase and during the rebound phase have been found previously by Guillaume (2012). Guillaume (2012) speculated that this effect could be caused by two different processing pathways, a fast one through the superior colliculus and a slower pathway via cortical areas such as the frontal eye field, resulting in saccades that were perturbed by the flash in the same way, just at two different points in time.

In the present study, the properties of early saccades started before the saccadic inhibition dip and the rebound phase saccades differed. Early saccades were clearly hyometric with unaltered peak velocities. The rebound saccades were indeed faster than regular saccades, however this effect was due to slightly declining peak velocities with increasing saccade latency. A comparison of the velocity profiles of the rebound saccades and regular saccades in the time window of the saccadic inhibition phase did not reveal any differences. This suggests that the rebound saccades were planned for an earlier start, delayed by the flash and were executed at their original velocity. Regarding the different results of these studies, one should note that Guillaume (2012) used a pattern mask to induce saccadic inhibition, whereas a flash or light mask was used in the present study. It is known that the origins and effects of pattern and light masking differ (see e.g., Breitmeyer, 2007).

The results of the present study agree with those by Edelman & Xu (2009), who used small, square distractors instead of flashes. They observed distractor-perturbed saccades with velocity profiles very similar to those of the present study. The results of both studies suggest that whenever the flash or distractor fell in the saccaddic dead time of a saccade, the programming of this saccade was not altered, but stopped in mid-flight. It seems plausible that just before saccadic activity is stopped, saccades that were already planned are completed as soon as possible.

But why did Buonocore, McIntosh & Melcher (2016) find early, hypometric saccades that were also faster than expected from the main sequence? In their experiments, participants made saccades along the horizontal midline of the screen to targets presented at one (experiments 1 and 3) or three (experiment 2) different eccentricities. That is, participants prepared saccades for just two different directions (left and right) and maximally three different amplitudes, resulting in maximally six different saccade plans. In the present experiment, participants always had to plan a saccade with a new and until target presentation unknown, direction and amplitude. If the difference between saccade plan reactivation, as in the experiments of Buonocore, McIntosh & Melcher (2016), and the from-scratch programming of a new saccade, as in the present experiment, results in different responses to perturbations such as flashes has not yet been directly tested. It is a limitation of the present study that it does not directly compare these two conditions.

However, it has been shown that saccadic reaction times decrease when repeated saccades are made to the same target (Dorris, Paré & Munoz, 2000), a finding that could explain the very early hypometric saccades found in the time bin of 20 ms to 40 after flash onset by Buonocore, McIntosh & Melcher (2016). In the present study, the earliest hypometric saccacades were found with the built-in algorithm of the eye tracker in the time bin from 34 ms to 54 ms after flash offset, which corresponds to 50.8 ms to 70.8 ms after flash onset.

## Conclusions

To conclude, the results of the present study suggest that saccades that need to be newly programmed and are started 34 ms to 54 ms after a 16.8 ms (one frame) flash, but before the saccadic inhibition dip in frequency, are actively stopped in mid-flight, but otherwise regular. Saccades that are started during the flash are not at all affected. Thus, for the type of saccades used in the present experiment, one could argue whether a saccade plan that is just stopped is sufficient to dispose of the idea of a saccadic dead time, or whether this would require that the saccade plan is modified.

## Additional Information and Declarations

### Data Availability

The following information was supplied regarding data availability: Dombrowe, Isabel. 2018. “Saccadic Inhibition in a Guided Saccade Task.” Open Science Framework. February 22. DOI: 10.17605/OSF.IO/PVBWM.
